# Preparation of Sourdoughs Fermented with Isolated Lactic Acid Bacteria and Characterization of Their Antifungal Properties

**DOI:** 10.3390/foods12040686

**Published:** 2023-02-04

**Authors:** Carla Lafuente, Jorge Calpe, Leonardo Musto, Tiago de Melo Nazareth, Victor Dopazo, Giuseppe Meca, Carlos Luz

**Affiliations:** 1 Department of Food Science and Toxicology, Faculty of Pharmacy, University of Valencia, Ave. Vicent Andrés Estellés s/n, 46100 Burjassot, Spain; 2 AgrotechUV Incubator, University of Valencia Science Park, St. Catedrático Agustín Escardino 9, 46980 Paterna, Spain

**Keywords:** *Lactiplantibacillus plantarum*, biocontrol, volatile organic compounds, *Pediococcus pentosaceus*, antifungal activity

## Abstract

Traditional sourdough is obtained using a mixture of flour and water stored at room temperature until acidification. Therefore, adding lactic acid bacteria (LAB) can improve the quality and safety of sourdough bread. Faced with this problem, four drying techniques—freeze-drying, spray-drying, low-temperature drying, and drying at low humidity—have been applied. Our goals were to isolate LAB strains with antifungal potential against *Aspergillus* and *Penicillium* fungi. The antifungal capacity was evaluated with agar diffusion, co-culture in overlay agar, and a microdilution susceptibility assay. In addition, the antifungal compounds generated in sourdough were analyzed. As a result, dried sourdoughs were prepared with *Lactiplantibacillus plantarum* TN10, *Lactiplantibacillus plantarum* TF2, *Pediococcus pentosaceus* TF8, *Pediococcus acidilactici* TE4, and *Pediococcus pentosaceus* TI6. The minimum fungicidal concentrations ranged from 25 g/L versus *P. verrucosum* and 100 g/L against *A. flavus*. A total of 27 volatile organic compounds were produced. Moreover, the lactic acid content reached 26 g/kg of dry product, and the phenyllactic concentration was significantly higher than the control. The *P. pentosaceus* TI6 exhibited a higher antifungal capacity in vitro and demonstrated a higher production of antifungal compounds compared to the other strains; therefore, further studies will evaluate the impact of this sourdough in bread manufacture.

## 1. Introduction

Microbial, chemical, enzymatic, or physical changes induce quality losses in some products. Fungal growth in particular is the most common cause of impairment in feed and food, and the toxicity of some species could generate health risks [1]. Some fungal genera, such as *Aspergillus* and *Penicillium,* can contaminate crops and produce toxic secondary metabolites, known as aflatoxins (AFB1, AFB2, AFG1, AFG2), produced by species of *Aspergillus,* and ochratoxins (Ochratoxin A (OTA)), produced by species of the genera *Aspergillus* and *Penicillium* [2,3]. Therefore, it is crucial to reduce microbial contamination in feed and food with efficient and secure techniques.

Biopreservation is any technique involving the use of microorganisms, extracts, or substances produced by them that extend a product’s shelf life and increase food safety [4]. Fermentation is one of the techniques used for the biopreservation of food, highlighting products such as bread, milk, wine, beer, meat, and cheese [5].

Lactic acid bacteria (LAB) are present in numerous foods and can be applied at an industrial level. They are therefore considered Generally Recognized as Safe (GRAS) organisms recognized by the Food and Drug Administration (FDA). Formerly, LAB were used in the fermentation process as a strategy to extend the shelf life of food [6]. In addition, due to the metabolites they produce, these organisms can inhibit the growth of pathogens [7]. Thanks to LAB, many safe foods have been produced, and they can currently be used as starter cultures to produce fermented products that have microbiological safety [8].

Many kinds of cereal, such as wheat, can be fermented using the LAB of the original dough, giving rise to the product known as sourdough [1]. These bacteria, primarily found in cereal fermentations, belong to the genera *Lactiplantibacillus*, *Enterococcus*, *Oenococcus*, *Bifidobacterium*, *Aerococcus*, *Carnobacterium*, *Lactococcus*, *Leuconostoc*, *Pediococcus*, *Streptococcus*, and *Weissella* [9,10,11,12,13].

One of the functions of using LAB as a sourdough ferment is as a bioconservant, since it helps prevent the growth of fungi [14]. The fermentation of the dough with LAB produces mainly lactic acid, which decreases the pH of the medium, inhibiting the growth of undesirable microorganisms [15]. Other active compounds of low molecular weight could be generated and act synergistically and, consequently, extend the product’s shelf life [4]. These fermentation products such as organic acids, aromatic compounds, and other metabolites, in addition to acidifying the environment and prolonging its conservation, provide better organoleptic, technological, and nutritional benefits to the final product [16,17].

Based on how sourdough is made, it is classified into three types. Type I sourdough is a fresh sourdough that is reactivated by adding a part of the previous dough. This type of dough requires care for the microorganisms to be active. Type II sourdough uses selected strains of microorganisms for the fermentation of the dough; it is a type of sourdough for industry use. Type III sourdough consists of elaborating a sourdough that is subsequently dried [18]. Due to the expensive and unstable preparation and maintenance of type I sourdough, drying is a solution for its stabilization since drying can extend the product’s shelf life until use. In addition, the cost of time and production of sourdough may be reduced [19].

In addition to the cost, there are other challenges in drying sourdoughs that concern the food industry. One of the challenges is the microbiological composition; according to Savkina et al. [20], sourdough has a rich flora of bacteria, but they are not only related to the fermentation process and are part of the natural contamination. Among them, it was evidenced that LAB strains isolated from sourdoughs could be used as a starter in sourdough fermentation. However, the drying methods applied should allow the cell viability that enables the fermentation of the final product. For this reason, this study aims to identify bacteria with an antifungal capacity that could increase the sourdough’s shelf life.

Several scientific articles showed different sourdough-drying techniques and pointed out the conclusions of how different drying methods have affected sourdough starters [18,19], among which drying in the oven, freeze-drying, and spray-drying are the most common techniques applied [21,22,23]. Even though some studies report the drying techniques such as freeze-drying and spray drying as suitable drying methods to preserve sourdough, there is a lack of information related to dry sourdough and its use in bread elaboration.

Considering the abovementioned issues, this study investigated the antifungal activity of isolated LAB from sourdough against five toxigenic fungi and their use as starter cultures in sourdough fermentation. In addition, we identified and quantified organic acids, phenolic acids, and volatile organic compounds produced in dry fermented sourdoughs. For this purpose, four methods were studied to obtain the dried sourdough powders: drying in the oven, freeze-drying, drying by lowering the humidity, and spray-drying.

## 2. Material and Methods

### 2.1. Chemicals

The culture media, potato dextrose broth (PDB), potato dextrose agar (PDA), de Man–Rogosa–Sharpe broth (MRS), agar de Man–Rogosa–Sharpe (MRS), Rose Bengal Chloramphenicol agar, and buffered peptone were acquired from Liofilchem Bacteriology Products (Roseto Degli Abruzzi, Italy). Deionized water (<18 MΩ/cm of resistivity) used to make the culture media was obtained using a Mili-Q purification system (Millipore, Bedford, MA, USA).

Solvents used in liquid chromatography were sulfuric acid, ethyl acetate, acetonitrile (ACN), and methanol from VRM Chemicals (VRM Chemical, Radnor, PA, USA). Sodium carbonate and sodium hydroxide were obtained from Fisher Scientific (Loughborough, UK). The Tween reagent and glycerol were from Sigma-Aldrich (St. Louis, MO, USA).

Different wheat flours of the Hacendado brand and Bezoya mineral water (Mercadona, Valencia, Spain) were used to elaborate the sourdoughs.

### 2.2. Microorganism Isolation and Fermented Cell-Free Supernatant (CFS)

A total of 40 LAB were isolated from different sourdoughs made with wheat flour: wheat flour (TN), whole wheat flour (TI), strength wheat flour (TF), and spelt flour (TE). The sourdoughs were diluted in a 1:10 (*w/w*) ratio with sterile peptone water 0.1% and plated on MRS agar, allowing the isolation of the LAB [24]. The plates were incubated at 37 °C, and after 24 h of incubation, different colonies were sown on new MRS plates to obtain a pure culture. The plates were incubated under anaerobic conditions using Mikrobiologie Anaerocult A (Merck Millipore, Rahway, NJ, USA). Afterward, the isolates were stored on MRS with 25% glycerol at −80 °C. Finally, the LAB were resuspended on MRS broth and incubated at 37 °C for 48 h before its application.

In order to obtain a cell-free supernatant (CFS), as described in Sung et al. [25] with modifications, the isolated strains fermented MRS broth, the fermented medium was centrifuged, and the supernatant was lyophilized. After the lyophilization of the metabolites produced by the microorganisms had been prepared, the CFS was obtained.

### 2.3. Qualitative In Vitro Evaluation of the Antifungal Activity of the Lyophilized MRS Medium Fermented with the Isolated Strains 

Once the microorganisms were isolated, a qualitative test was carried out in order to rule out those strains with lower antifungal capacity. The fungi selected for this initial screening were *Aspergillus flavus* ISPA 8111, *Penicillium expansum* CECT 2278, *Penicillium verrucosum* VTT 0184, *Penicillium digitatum* CECT 2954, and *Penicillium commune* CECT 20767. The screening of bacteria was performed using an agar diffusion assay described by Luz et al. [26] with modifications.

The agar diffusion method was used to study fermented CFS’s antifungal capacity against five fungi of the *Penicillium* and *Aspergillus* genera. First, the fungus was sown with a swab in PDA plates under sterile conditions. Then, in the same plates, the agar was perforated with pipette tips, leaving holes in which 100 μL of freeze-dried CFS was introduced in a concentration of 500 g/L. Then, the plates were incubated at 25 °C for 48 h. Finally, the inhibition halo was measured to determine the CFS antifungal activity.

### 2.4. Morphological Characterization and Identification of the LAB

Morphological characterization with Gram staining was carried out for the isolated strains that present the most significant antifungal activity in the qualitative test. Consequently, the strains identified were *Lactiplantibacillus plantarum* TN10, *Lactiplantibacillus plantarum* TF2, *Pediococcus pentosaceus* TF8, *Pediococcus acidilactici* TE4, and *Pediococcus pentosaceus* TI6.

Subsequently, the strains were identified following the protocol of Dopazo et al. [27], using the extended direct transfer method. Mass spectrometry, matrix-assisted laser desorption/ionization time-of-flight mass spectrometry (MALDI-TOF/MS), was used to identify microorganisms creating a spectrum based on the protein profile; each protein profile is unique to each species. Thus, the MALDI-TOF/MS technique was carried out using a Microflex L20 mass spectrometer equipped with an N2 laser. Moreover, all the spectra were acquired in positive linear ion mode. The acceleration voltage was 20 kV. The spectra were acquired as the sum of 240 shots per target, and the mass range used for analysis was 2000–20,000 Da.

### 2.5. Qualitative In Vitro Evaluation of the Antifungal Activity of the LAB

As described in Luz et al. [7], the overlay assay was performed to study the antifungal activity of the five LAB selected against *A. flavus* ISPA 8111, *P. expansum* CECT 2278, *P. verrucosum* VTT 0184, *P. digitatum* CECT 2954 and *P. commune* CECT 20767. After 24 h of growth of each microbial suspension, 20 μL of bacteria was inoculated in the middle of the MRS-A plate and incubated for 72 h at 37 °C. After that, the plates were covered with 20 mL of PDA with a 5% solution of fungal spores of each fungus. Once the test was completed, the plates were incubated at 25 °C for 48 h.

### 2.6. Quantitative In Vitro Evaluation of the Antifungal Activity of the Lyophilized MRS Medium Fermented with the Five Strains

The assay of the minimum inhibitory concentration (MIC) and minimum fungicidal concentration (MFC) was performed as described by Mandal et al. [28].

Before the test, 2 g of the lyophilized fermented MRS broth was resuspended and inoculated in 10 mL of sterile water, and a fungal spore solution of 10^5^ conidia/mL was prepared. Microplates with 96 wells were ordered for the MIC assay because the negative and positive controls were located in the first and second columns. The controls were performed to verify that the study was carried out correctly. The negative control contained 200 μL of sterile PDB, and the positive contained 100 μL of sterile PDB culture medium and 100 μL of the 10^5^ CFU/mL fungal spore suspension. In the rest of the wells, 100 μL of sterile PDB was added, and a serial dilution of CFS was performed. Subsequently, 100 μL of the spore solution (10^5^ conidia/mL) was inserted into each well, obtaining a final concentration of 5 × 0^4^ conidia/mL. Finally, the microplates were incubated at 25 °C for 72 h.

The MIC was determined as the concentration that inhibited the visual fungal growth. After that, the MIC and higher concentrations were resown on PDA plates and incubated at 25 °C for 72 h in order to determine the MFC. Thus, the MFC results were established as the lowest CFS concentration that entirely avoids fungal growth after reculture.

### 2.7. Analysis of Volatile Organic Compounds (VOCs) of MRS Fermented with the LAB

The VOCs determination in the samples was performed through a microextraction in solid-phase of the headspace (HS-SPME) and its posterior analysis with gas chromatography with a single-quadrupole mass spectrometer detector (GC/MS) following the methodology described by Luz et al. [29] with modifications. 

The analyzed samples were prepared by adding 1 g of lyophilized CFS of each LAB to 5 mL of MiliQ water in a vial. The vials were incubated in a 50 °C bath for 45 min under constant agitation. The analyzed VOCs of the samples were extracted through a DVB/C-WR/PDMS coated SPME fiber (80 µm × 10 mm) (Supelco, Bellafonte, PA, USA), and the analysis was carried out using gas chromatography with a single-quadrupole mass spectrometer detector (GC/MS). The fiber was introduced into an Agilent 7890A gas chromatograph coupled to an Agilent 7000A triple quadrupole mass spectrometer equipped with an electronic impact source (EI). The desorption was performed at 250 °C for ten minutes, and fiber injection was carried out in spitless mode. HP-5MS (30 m × 0.25 mm, 0.25 µm) (J&W Scientific, Folsom, CA, USA) was the column used for the chromatographic separation. Regarding the temperature reached, a temperature ramp was programmed. First, the program started at 40 °C for 2 min and increased to 160 °C at 6 °C/min after boosting the temperature to 260 °C at 10 °C/min and keeping it constant for 40 min. The flow of the carrier gas, 99.99% helium, was 2,5 mL/min. The detection of the compounds was carried out in a range *m/z* of 40–50 Da in full scan mode. The compounds were identified through the NIST Atomic Spectra Database version 1.6 (Gaithersburg, MD, USA) using 95% spectral similarity. The linear retention index (LRI) was calculated regarding the time retention of an alkane solution (C8-C20) analyzed with the same conditions as the samples. The proportion of VOC in the sample was calculated by dividing the analyte area by the total area.

### 2.8. Dry Sourdough Elaboration

The sourdough elaboration was performed as described by Wu et al. [30], with modifications. The five LAB strains were activated in MRS broth at 37 °C for 24 h, and then they were cultured for a second time in 100 mL of MRS broth for 24 h. The obtained suspension was centrifuged at 7700× *g* at 4 °C for 10 min. After centrifuging the samples, the precipitate was resuspended in 125 mL of sterile mineral water and mixed with 100 g of sterile wheat flour in order to elaborate an inoculum of 10^7^ LAB CFU/g of sourdough.

Next to the sourdough preparation in aseptic conditions, the doughs were fermented and fed using the back-lopping method every 48 h, favoring the continuous fermentation of the product [31]. The doughs were kept at 25–28 °C until they were refreshed with 50 mL of sterile mineral water and 50 g of sterile wheat flour. After seven days of fermentation, the dough was extended and dried to obtain a valuable dry sourdough for bread making. Four drying techniques were employed to dry these sourdoughs.

A control sourdough was prepared following the same steps. However, the control sourdough did not receive LAB inoculum so that sterile wheat flour was mixed with sterile mineral water.

#### 2.8.1. Freeze-Drying Technique

To freeze-dry the sample, 40 g of the sourdoughs was aliquoted and kept at −40 °C for 24 h for the subsequent lyophilization [22]. The lyophilization process was carried out using the freeze-dryer FreeZone 2.5 L Labconco (Kansas, MO, USA) and vacuum freeze-drying at 15 Pa for 50 h. The resulting powder was stored in sterile containers.

#### 2.8.2. Spray-Dry

The spray-drying equipment OLT-SD8000B (XIAMEN Ollital Technology Co., Ltd., Xiamen, Fujian, China) was used for this drying. First, 40 g of each sourdough was taken and diluted in a proportion of distilled water 1:2 (*m*/*v*) [22]. The drying medium was air. Spray-drying experiments were carried out at 100.0 °C inlet air temperature and 55.0 °C outlet air temperature. The parameter of fan speed was 70.0, and an 8 mL/min liquid flow rate was set. The resulting powder was stored in sterile containers.

#### 2.8.3. Low-Humidity Drying

Another technique was drying by decreasing the humidity, which is drying at a low temperature (20 °C). It is carried out in conditioned storage equipment, which only uses electrical energy to move the air with a fan. The thermal energy provided evaporates the moisture and eliminates it to the outside. Aliquots of 40 g of the sourdoughs were taken and dried at 20 °C for 2 h in a dryer that lowers the humidity. The resulting powder was stored in sterile containers.

#### 2.8.4. Low-Temperature Drying

In this method, 40 g of the sourdoughs were dried in an oven at a low temperature, 40 °C for 24 h, as detailed in the study of Ertop et al. [22]. The oven used was a Buffalo Appliance convection oven. The resulting powder was stored in sterile containers.

### 2.9. Determination of pH, Viable Cell Count, and Weight during Sourdough Fermentation

The viability of bacteria cells and pH were measured at three time points: at time zero (t_0_), in the final stage of sourdough fermentation, and after sourdough drying. For the cell count, 1 g of each sourdough was diluted with peptone water (0.1%), plated on MRS agar, and then maintained at 37 °C for 48 h [7]. The pH was measured with a solid pH meter G-PH7V-3 VIO with electrode XS 201TN (Labprocess, Barcelona, Spain). Finally, the weight of the dough after drying was also measured in order to see the yield of the sourdoughs.

### 2.10. Determination of Organic Acids in Dried Sourdoughs

Determining organic acids in the dry sourdoughs was carried out following the procedure described by de la Fuente et al. [32] with modifications. Sourdough samples were diluted 1:20 with deionized water and homogenized using an Ultra Ika T18 Ultraturrax (Staufen, Germany). Then the samples were filtered using a cellulose filter membrane with 0.22 µm pore size before its introduction into the high-performance liquid chromatography (HPLC) system (Agilent 1100 Series HPLC System, Santa Clara, CA, USA). The HPLC was fitted out with a Jasco PU-4180 pump (Easton, Maryland, USA), a reverse phase column Rezex ROA-Organic Acid (150 mm × 7.8 mm) (Phenomenex Inc., Torrance, CA, USA), a diode array detector Jasco MD-4015 (Easton, MD, USA), and 20 µL of the sample injection. The mobile phase was a solution of water and sulphuric acid 0.005 M with a flow of 0.8 mL/min for 10 min at 40 °C. The detectors’ wavelength for quantifying organic acids was established at 214 nm. The calibration curve was carried out using acetic acid and lactic acid diluted in water at concentrations from 0 to 1000 mg/L, and the samples were tested in triplicate. Results were expressed in g/Kg, and the software used for data collection was ChromNav 2.0 HPLC (Jasco, Easton, MD, USA).

### 2.11. Determination of Phenolic Compounds in Dried Sourdoughs

The phenolic acids of the sourdough were studied through the procedure described in Dopazo et al. [33] with modifications. First, the sourdough samples were purified through QuEChERS extraction. A 15 mL tube with 4 g of MgSO_4_, 1 g of NaCl, and 5 mL of ethyl acetate with 1% formic acid (*v*/*v*) was prepared. Next 5 mL of each sourdough sample diluted 1/4 with deionized water was added into the tubes and they were mixed with a vortex. After that, the tubes were incubated on ice for a few minutes until they were centrifuged at 7700× *g* for 10 min and 4 °C. The resulting supernatant of each tube was transferred into a new 15 mL tube before drying using the TurboVap to evaporate the supernatant under nitrogen flow at −20 °C.

After obtaining the purified samples, they were resuspended in 1 mL of water with acetonitrile 90:10 (*v*/*v*). The purified solution was filtered in vials, and the analysis was performed using LS Agilent 1200 system (Santa Clara, CA, USA). The column employed was Gemini C18 (50 mm × 2 mm), 100 Å, and 3 μm particle size. The mobile phase used was water as solvent A and acetonitrile as solvent B, both acidified with 1% formic acid. The elution gradient selected was at 0 min time 5% of solvent B; 30 min 95% B; 35 min 5% B, at a flow of 3 mL/min. Finally, the injected volume was 20 μL.

Mass spectrometry was performed using Q-TOF-MS 6540 Agilent Ultra High-Definition Accurate Mass equipped with an electrospray ionizer Agilent Dual Jet Stream Dual JS ESI. The equipment was programmed with negative ionization mode. The conditions were that the drying gas was set at 350 °C and its flow rate was 8 mL/min, the capillary voltage was 3.5 kV, the voltage fragmentation was 175 V, the nebulizer pressure was 30 psi and the scan range, *m/z*, was 20–380. The collision energies used were 10, 20, and 40 eV to carry out the MS/MS assay. Data collection was administrated using Masshunter Qualitative Analysis Software (version B.08.00) (Santa Clara, CA, USA).

### 2.12. Statistical Analyses

All analyses were performed in at least triplicate to confirm the reproducibility of results. The data are expressed in mean values and standard deviation. InfoStat version 2020 (Universidad Nacional de Córdoba, Córdoba, Argentina) was the software used for the statistical analysis. The two-way ANOVA was performed to assess data, and the significant differences between samples and treatments were analyzed using Tukey’s multiple comparison test. Moreover, the *t*-test was performed to compare two groups. The significance level was adjusted to a *p*-value ≤ 0.05 for all analyses.

## 3. Results and Discussion

### 3.1. Isolation, Screening, and Identification of the LAB

A total of 40 microorganisms isolated from different sourdoughs were evaluated to determine their antifungal capacity. The diffusion agar assay was performed to determine which strains presented the highest antifungal activity. As shown in Table 1, five strains stand out for their antifungal capacity. Most CFS had fungicidal activity against fungi of the genera *Aspergillus* and *Penicillium.* However, the fungi *Penicillium digitatum* shows resistance to the CFS of the isolated strains.

According to the halo of inhibition of fungal growth, the activity of the strains TN10, TF2, TF8, TE4, and TI6 was highlighted. These five strains were selected for the continuation of the study. Therefore, strains were identified using the MALDI-TOF MS method and classified with the MBT 7854 and MBT 7311_ RUO database (Bruker Daltonics, Billerica, Massachusetts, USA). This method verified the identity of the microorganisms at the species level: *Lactiplantibacillus plantarum* TN10, *Lactiplantibacillus plantarum* TF2, *Pediococcus pentosaceus* TF8, *Pediococcus acidilactici* TE4, and *Pediococcus pentosaceus* TI6. The identifications of the species with a Log score superior to 2 were considered. Henceforth, the acronyms TE4, TF2, TF8, TI6, and TN10 will be applied to refer to their respective strain. Moreover, it is essential to emphasize that LAB are recognized as safe by the EFSA, and their presence in food may be beneficial.

Against this background, several studies have reported the presence of *Lactiplantibacillus* and *Pediococcus* in sourdough and their use as biopreservative agents [9]. Minervini et al. [11] demonstrated that the nera *Lactiplantibacillus*, *Lactococcus*, *Streptococcus*, *Pediococcus,* and *Enterococcus* are endophytic microorganisms of sourdough and originated in cereal grain.

During fermentation, the LAB population of sourdough is responsible for the final product’s beneficial properties. This group of microorganisms is relevant as starter cultures in fermented food to preserve them and guarantee their safety. Furthermore, isolated LAB from sourdough are suitable for preserved sourdough because they are adapted to these food conditions [34].

Studies such as that of Sadeghi et al. [35] proved that compounds generated in LAB fermentation result in an impediment against mold contamination. The study evidenced the opposite activity of *P. pentosaceus* against fungi of the genus *Aspergillus*. Furthermore, the authors discovered that *P. pentosaceus* produced antifungal preservative compounds with novel applications in different areas of interest.

Other studies, such as that of de Melo et al. [36], showed the antifungal capacity of the *L. plantarum* to inhibit the growth of different fungi, including *A. flavus*, *F. graminearum*, *F. cerealis,* and *F. verticillioides*.

### 3.2. Qualitative and Quantitative In Vitro Evaluation of the Antifungal Activity of the LAB

The antifungal activity of the LAB was evaluated using the overlay method against fungus of the genera *Aspergillus* and *Penicillium*. It is worth noting that the strain with the highest antifungal effect was *P. pentosaceus* TI6; the antifungal activity results are plotted in Table 2.

Moreover, the MIC-MFC concentrations were evaluated in order to quantify the antifungal capacity of the CFS of isolated LAB strains, as summarized in Table 3. The *P. pentosaceus* TF8 strain gave rise to the compounds with the most significant antifungal effect at its lowest CFS concentrations. In addition, we could highlight the activity of the microorganisms *L. plantarum* TF2 and *P. pentosaceus* TI6. The TF2, TF8, and TI6 strains give the best MIC-MFC values, reaching minimum fungicidal concentrations of 25 g/L versus *P. verrucosum* and about 100 g/L against *A. flavus*.

As expected, *P. digitatum* is the fungus that presents more resistance, for removing their spores requires the highest dose of ferments produced by the strains. However, visually it is more complex to see their growth since the MFC of all bacteria does not exceed 50 g/L.

The quantitative evaluation of the antifungal activity of the selected strains evidenced the antifungal potential of these isolated LAB as sourdough ferment. Previous studies have evaluated the antifungal capacity of isolated LAB from sourdough. For instance, Sadeghi et al. [35] showed that a *P. pentosaceus* strain prevented mycelial growth of *Aspergillus* fungi. Knowing the mode of action of the LAB strains can be a biocontrol procedure in its application, taking into account that the growth stage of conidia is the most susceptible stage. 

Contemplating the fungal inhibition, this study investigated the antifungal compounds generated during sourdough fermentation and after it was dried. 

### 3.3. Determination of VOCs of MRS Fermented with the LAB

**Figure 1:**
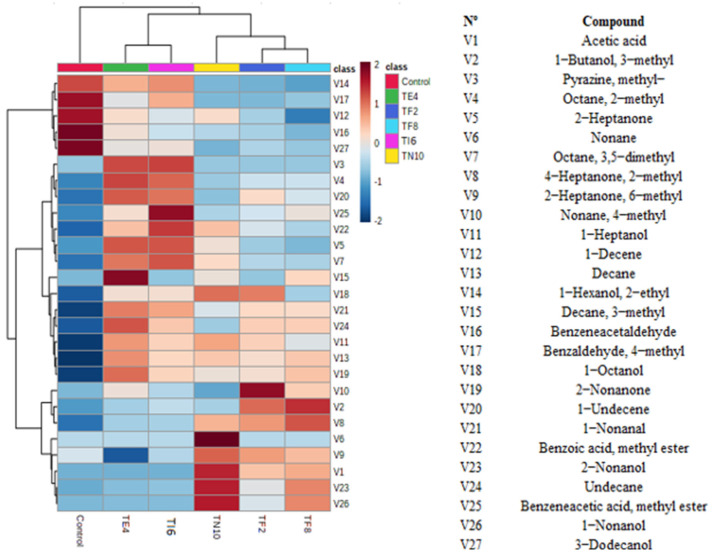
Heatmap of the abundance of the volatile organic compounds produced by *Lactiplantibacillus plantarum* TN10, *Lactiplantibacillus plantarum* TF2, *Pediococcus pentosaceus* TF8, *Pediococcus acidilactici* TE4 and *Pediococcus pentosaceus* TI6 in the cell-free supernatant.

The VOCs in lyophilized CFS fermented with the LAB are shown in Figure 1. Twenty-seven VOCs were evaluated, and these generated compounds can be classified as acids, alcohols, aldehydes, alkanes, alkenes, esters, ketones, and pyrazines.

In the samples, mainly alcohols, aldehydes, alkanes, alkenes, and pyrazines can be found. The VOCs determined may be due to the metabolites generated by the LAB during the fermentation. The volatile compounds generated by microorganisms developed in the sourdough give rise to enzymes that act in the hydrolysis of the sugars present in the flour, producing mainly ethanol, CO_2_, and volatile acids. The generation of these volatile compounds produces a series of characteristic qualities that also extend the conservation of the product [37].

As seen in Figure 1, the most striking point from the heatmap comparison was the difference in volatile compounds between the samples. The compounds 1-hexanol-2-ethyl, benzaldehyde 4-methyl, 1-Decene, benzeneacetaldehyde, and 3-Dodecanol were the VOCs determined in most abundance in the control sample. Moreover, benzeneacetic acid methyl ester, benzoic acid methyl ester, decane 3-methyl, nonane, 1-Butanol 3-methyl, acetic acid, nonane 4-methyl, pyrazine methyl, 2-nonanol, and 1-nonanol were the compounds in most abundance in the samples fermented with the LAB.

The volatile profile of the CFS of the MRS fermented with *P. pentosaceus* TI6 presents a more outstanding content of benzeneacetic acid, methyl ester, pyrazine, methyl and benzoic acid. Esters were significant compounds involved in fermented products’ flavor [38]. The Wang et al. [39] study reported that the volatile compound benzeneacetic acid presents antifungal capacity against fungi *Botrytis cinerea*, *Glomerella cingulate*, *Phytophthora drechsleri* Tucker, *Penicillium citrinum*, *Penicillium digitatum,* and *Fusarium oxysporum*. Likewise, Su et al. [40] studied the inhibitory effects of the pyrazine ester derivates showing great antifungal capacity against the fungi *F. graminearum*, *F. oxysporum*, *R. solani, F. moniliforme*, and *P. nicotianae*.

The volatile profile of the CFS of the MRS fermented with *L. plantarum* highlights the content of acetic acid, alcohol as 1-nonanol and 2-nonanol, and alkanes such as nonane and 4-methyl nonane.

Studies by Zhang et al. [41] have shown that 1-nonanol is a primary component of cereal volatiles and has an effect against the pathogen *A. flavus,* preventing the growth of fungicidal spores and mycelium and being a potential mechanism of biopreservation. 

In general, qualitative and quantitative results confirmed a positive correlation between the volatile compounds generated, such as acids and alcohols, and the fungal inhibition. Moreover, Digaitiene [42] described that the interactions between LAB and fungi resulted in the production of organic acids and other molecules that, once associated with volatile compounds, could increase the antifungal capacity of these microorganisms.

### 3.4. Determination of pH, Viable Cell Count, and Weight during Sourdough Fermentation

As expected, the pH of the samples changed during sourdough fermentation. The LAB in sourdough fermentation hydrolyzed glucose to generate lactic acid and acidify the medium [43] so that the pH of the sourdoughs fermented by LAB decreased compared to the control sourdough, as can be seen in Table 4.

The reduction in the pH, obtaining a final pH of 3.45–3.80, showed significant sourdough development, as Siepmann et al. [44] reported. The sourdoughs fermented with the strains *L. plantarum* TN10 and *P. pentosaceus* TI6 had the largest pH difference between the time points. Previous studies such as that of Fu et al. [45] showed that the strain *P. pentosaceus* acidified the medium quickly compared to the strain *L. plantarum* and reported both strains as good starters in sourdough fermentation.

Regarding microbiological analysis, the number of viable microorganisms grew during the sourdough fermentation. Table 5 shows the viability of the LAB before and after sourdough fermentation and in the dried sourdough. The sourdoughs fermented with *L. plantarum* TN10, *P. pentosaceus* TI6, and *P. acidilactici* TE4 showed the highest count of cells before and after fermentation, reaching 8.85 to 10.60 log CFU/g.

In dried samples, the viability of the microorganisms decreased in comparison to the fermented sourdoughs due to the conditions of the drying techniques used. Ertop et al. [22] studied the decrease in the viability of LAB after drying sourdough in an oven at low temperature compared to other drying techniques such as lyophilization or spray-drying. Indeed, this study shows less viability of microorganisms after drying in the oven than by spray-drying or freeze-drying.

The powders were weighed after different drying techniques to determine the yield of the dough. Sourdough fermented with *P. pentosaceus* TI6 and dried in an oven at 40 °C had the best yield (Table 6).

### 3.5. Determination of Organic Acids in Dried Sourdough

Organic acids are the principal metabolites produced by LAB, and their action has antifungal activity due to the synergy between these organic acids, particularly lactic acid [46]. Lactic acid produced in the sourdoughs by the LAB fermentation have antimicrobial properties with an impact against unfavorable spoilage and decreasing the pH of the medium [47]. Table 7 summarizes the results obtained for determining the lactic acid in the dry sourdoughs and the control sample.

The five strains produced lactic acid due to their metabolism. As expected, each sourdough had a higher lactic acid content than the control sourdough. Usually, concentrations of 26 g of lactic acid per kg of the dry product were reached. Otherwise, there were differences between each culture starter of the sourdough and the dry technique used in producing lactic acid. The highest production of acid was in spray-dry sourdoughs fermented with *L. plantarum* TN10 with 26.3 g/kg and *P. acidilactici* TE4 with 20.4 g/kg, while the lowest production of lactic acid was in the sourdoughs dried at low humidity. The high concentration of lactic acid in the spray-dried samples may be due to a higher concentration of the compounds since much less powder was obtained.

According to the literature, several studies report that lactic acid’s effect correlates with the inhibitory activity against fungi, as seen in Luz et al. [7]. In addition, the presence of lactic acid in the sourdough can ensure its microbiological safety and prolong the final product’s shelf life if the organic acid remains undissociated in the fermented product [48]. As the significant LAB metabolite, lactic acid reduces pH, and the undissociated acid collapses the electrochemical proton gradient. Studies on the effect of LAB in conjunction with fungi are complicated by considering the observed differences in sensitivity among mold species to organic acids. These differences may be related to the ability of fungi to change cellular metabolism in response to stress conditions such as high concentrations of organic acids. Specifically, organic acids generated by LAB exert their antimicrobial effect by entering cell membranes in their undissociated form, resulting in a decrease in intercellular pH and interruption of metabolic processes. [35].

### 3.6. Determination of Phenolic Compounds in Dried Sourdoughs

Phenolic compounds are secondary compounds usually produced by cereals and represent a group of phytochemical substances that can be divided into different types of components regarding chemical structure: phenolics, flavonoids, stilbenes, lignans, and tannins [49].

**Figure 2:**
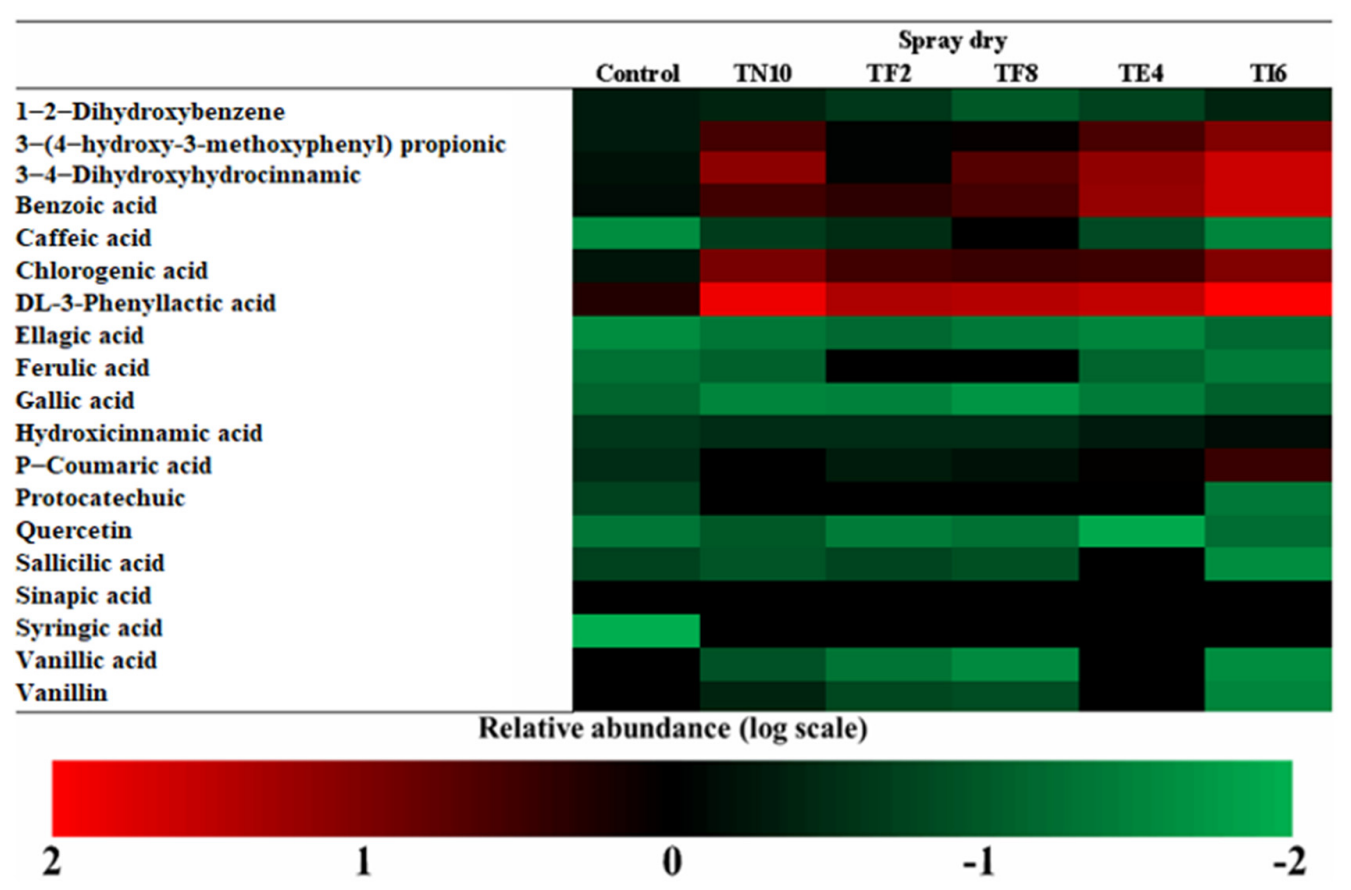
Determination of the phenolic compounds in atomized sourdoughs fermented with *Lactiplantibacillus plantarum* TN10, *Lactiplantibacillus plantarum* TF2, *Pediococcus pentosaceus* TF8, *Pediococcus acidilactici* TE4, and *Pediococcus pentosaceus* TI6.

**Figure 3:**
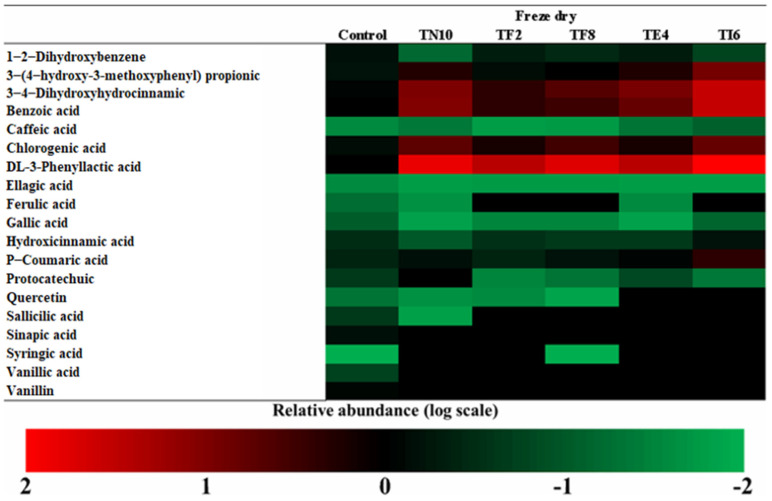
Determination of the phenolic compounds in lyophilized sourdoughs fermented with *Lactiplantibacillus plantarum* TN10, *Lactiplantibacillus plantarum* TF2, *Pediococcus pentosaceus* TF8, *Pediococcus acidilactici* TE4, and *Pediococcus pentosaceus* TI6.

**Figure 4:**
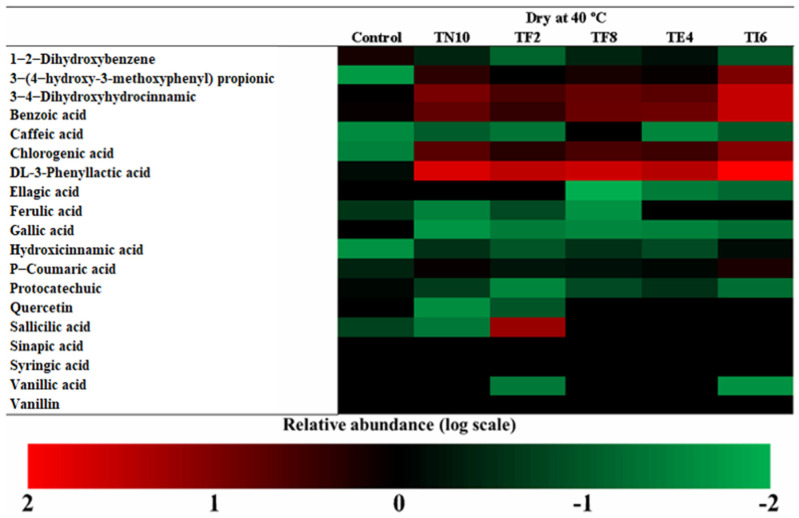
Determination of the phenolic compounds in sourdoughs fermented with *Lactiplantibacillus plantarum* TN10, *Lactiplantibacillus plantarum* TF2, *Pediococcus pentosaceus* TF8, *Pediococcus acidilactici* TE4, and *Pediococcus pentosaceus* TI6 and dried at 40 °C.

**Figure 5:**
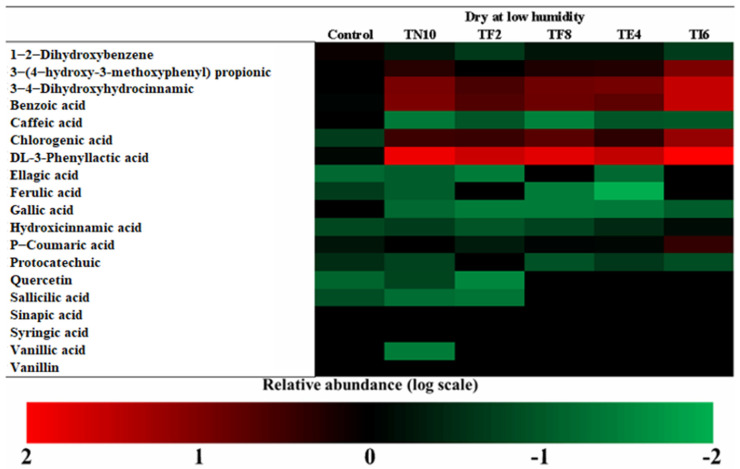
Determination of the phenolic compounds in sourdoughs fermented with *Lactiplantibacillus plantarum* TN10, *Lactiplantibacillus plantarum* TF2, *Pediococcus pentosaceus* TF8, *Pediococcus acidilactici* TE4, and *Pediococcus pentosaceus* TI6 and dried at low humidity.

The more abundant acids in dry sourdoughs were DL-3-Phenyllactic acid, benzoic acid, and 3,4-dihidroxihodrocinamic acid, as seen in Figure 2, Figure 3, Figure 4 and Figure 5.

Amborabé et al. [50] report that the production of these compounds has a relation with the highest antifungal activity. Regarding the results of the determination of the antifungal activity obtained in the in vitro assays, the strains are good antifungal agents due to the phenolic compounds produced by the LAB, among others. Lavermicocca et al. [51] studied the action of phenyllactic acid as an antifungal compound and the presence of phenyllactic acid in the samples has been related to the activity against fungi. However, it cannot be related to the production of phenyllactic acid with the antifungal capacity of the strain [15]. 

Otherwise, studies such as those of Dopazo et al. [27] and Axel et al. [14] reported the relationship of the presence of phenyllactic acid to fungal inhibition. Both studies elucidated the synergy of the organic acids and phenolic compounds as antifungal compounds.

The results highlight the sourdoughs fermented with *P. pentosaceus* TI6, which produces the maximum amount of phenolic compounds, such as DL-3-phenyllactic acid, benzoic acid, 3,4-dihidroxihidrocinamic acid, 3-(4-hidroxy-3-metoxyphenyl) propionic acid, and chlorogenic acid. It is noted that the phenolic compounds determined in the dry sourdoughs included 3,4-Dihydroxyhydrocinnamic acid, p-coumaric acid, phenyllactic acid, 3-(4-hydroxy-3-methoxyphenyl) propionic acid, and benzoic acid, similar to those of the study of Yépez et al. [52] produced by LAB strains isolated from Andean fermented products. 

Furthermore, in vitro studies such as that of Koistinen et al. [53] showed that some LAB strains metabolize the primary phenolic acids, giving rise to bioactive compounds with antioxidant, antimicrobial, and anticarcinogenic effects. The acidic environment during fermentation causes enzymes to hydrolyze phenolic compounds, making them active and generating antifungal compounds against fungal growth, providing the sourdough with a higher value and shelf life [54].

Thus, studying antifungal compounds, their action mechanism, and the discovery of appropriate applications can lead to a novel use of these LAB, especially *P. pentosaceus* TI6, to control the fungal contamination in fermented cereal products and increase their value. However, previous studies focused on the phenolic profile of sourdoughs, while the application of different drying techniques is still in development. Therefore, the literature lacks information about the combination of both techniques.

## 4. Conclusions

The main purposes of this study were to determine the antifungal activity of the isolated strains from different sourdoughs and evaluate the fungal inhibition against fungi of the genera *Aspergillus* and *Penicillium*. The in vitro results showed that five LAB strains evidenced significant antifungal activity, and the profile of VOCs revealed the production of volatile antifungal compounds. The strains *L. plantarum* TN10, *P. pentosaceus* TI6, *P. acidilactici* TE4, *L. plantarum* TF2, and *P. pentosaceus* TF8 were the LAB selected for the fermentation of the different sourdoughs. Next, we investigated the generation of dried sourdough’s antifungal compounds maintained after four drying techniques. The drying of the sourdough resulted in a series of changes in its microbiota; consequently, drying the dough lowered the LAB count. However, the viable cells remained adequate for proper fermentation for their future application in bread making.

Dried fermented sourdough showed a higher lactic acid content, reaching values of 26 g of lactic acid/kg of dry product, and a higher phenolic profile than the control group. Despite the concentration of organic and phenolic acids depending on the strain assayed, all treatments showed higher values than the control group. Nevertheless, comparing the strains, *P. pentosaceus* demonstrated higher values of antifungal compounds regardless of the drying technique. Moreover, dried sourdough fermented using the *P. pentosaceus* TI6 strain and dried at 40 °C were the optimal conditions for future bread-making applications, because the resulting powder had the best results in terms of antifungal activity, the viability of the microorganism, and yield of the product’s weight compared with other sourdoughs. It is important to consider this for later applications at an industrial level.

In short, although the study of dry sourdoughs is still under development, up to now, we have obtained relevant results that allow us to elaborate sourdoughs that can achieve better results than conventional ones. Therefore, subsequent studies will focus on dry sourdough as an ingredient in the preparation of bread; moreover, the antifungal effect and the extension of the shelf life of these products should be performed.

## Figures and Tables

**Table 1:** Diffusion agar results of the different strains against fungi *Penicillium expansum*, *Penicillium digitatum*, *Penicillium commune*, *Penicillium verrucosum,* and *Aspergillus flavus*. Results are expressed as ‘+’ if the inhibition is of 1–2 mm, ‘++’ 2–5 mm, ‘+++’ 5–10 mm, and ‘++++’ if the halo inhibition > 10 mm.

Fungi	Strains																															
	TN1	TN2	TN3		TN4		TN5			TN6		TN7				TN8			TN9		TN10				TN11			TN12			TN13	
*P. expansum*	++	++	++		++		+			+++		+++				+			++		+++				++			++			++	
*P. digitatum*	-	-	-		-		-			-		-				-			-		-				-			-			-	
*P. commune*	++	+++	++		++		++			++		++				++			++		+++				++			++			++	
*P. verrucosum*	+++	+++	+++		+++		+++			+++		+++				+++			+++		++++				+++			++++			+++	
*A. flavus*	+	+	+		+		-			+		+				+			+		++				++			++			+	
Fungi	Strains																															
	TI1	TI2	TI3		TI4		TI5		TI6		TI7				TI8		TI9			TI10		TI11		TI12			TI13			TI14		TI15
*P. expansum*	++	++	++		++		++		+++		++				++		++			++		++		++			+			++		++
*P. digitatum*	-	-	-		-		-		-		-				-		-			-		-		-			-			-		-
*P. commune*	++	++	+++		++		++		+++		++				++		++			++		++		++			+			++		++
*P. verrucosum*	+++	+++	+++		+++		+++		+++		+++				+++		+++			+++		+++		++++			+++			+++		+++
*A. flavus*	+	+	+		+		+		+		+				+		+			+		+		+			-			+		+
Fungi	Strains																															
	TE1		TE2			TE3			TE4				TE5					TE6			TE7					TE8			TE9		TE10	
*P. expansum*	++		++			+++			+++				++					++			++					++			++		++	
*P. digitatum*	-		-			-			-				-					-			-					-			-		-	
*P. commune*	++		++			++			++				++					++			++					+++			+++		+++	
*P. verrucosum*	+++		+++			+++			+++				+++					+++			+++					+++			+++		+++	
*A. flavus*	+		+			+			++				++					+			+					+			+		++	
Fungi	Strains																															
	TF1			TF2				TF3						TF4					TF5				TF6				TF7				TF8	
*P. expansum*	++			+++				++						++					+++				+++				++				+++	
*P. digitatum*	-			-				-						-					-				-				-				-	
*P. commune*	+++			+++				++						++					++				++				+++				+++	
*P. verrucosum*	+++			++++				++++						+++					++++				+++				+++				++++	
*A. flavus*	-			++				-						-					-				-				+				++	

Strains were isolated from TN: wheat flour, TI: whole wheat flour, TF: strength wheat flour, and TE: spelt flour.

**Table 2:** Overlay assay results of the five lactic acid bacteria selected against fungi *Penicillium expansum*, *Penicillium digitatum*, *Penicillium commune*, *Penicillium verrucosum,* and *Aspergillus flavus*. Results are expressed as ‘+’ if the inhibition is of 1–2 mm, ‘++’ 2–5 mm, ‘+++’ 5–10 mm.

Fungi	Strains *				
	TN10	TF2	TF8	TE4	TI6
*P. expansum*	+	+	+++	+	+++
*P. digitatum*	-	-	-	-	+
*A. flavus*	-	++	+	-	++
*P. commune*	+	+++	+	-	+++
*P. verrucosum*	+++	+++	+++	+++	+++

* Strains: Lactiplantibacillus plantarum TN10, Lactiplantibacillus plantarum TF2, Pediococcus pentosaceus TF8, Pediococcus acidilactici TE4, and Pediococcus pentosaceus TI6.

**Table 3:** Minimum inhibitory concentration (MIC) and minimum fungicidal concentration (MFC) results of the metabolites produced by lactic acid bacteria against *Penicillium expansum*, *Penicillium digitatum*, *Penicillium commune*, *Penicillium verrucosum,* and *Aspergillus flavus*.

Fungi	Strains *									
	TN10		TF2		TF8		TE4		TI6	
	MIC	MFC	MIC	MFC	MIC	MFC	MIC	MFC	MIC	MFC
*A. flavus*	50.0	100.0	50.0	100.0	50.0	100.0	50.0	100.0	50.0	100.0
*P. expansum*	50.0	100.0	25.0	50.0	25.0	25.0	50.0	50.0	25.0	50.0
*P. digitatum*	50.0	>200.0	25.0	>200.0	25.0	>200.0	50.0	>200.0	50.0	>200.0
*P. commune*	50.0	100.0	50.0	100.0	50.0	100.0	50.0	100.0	50.0	100.0
*P. verrucosum*	25.0	25.0	12.5	25.0	12.5	25.0	25.0	50.0	12.5	25.0

* Strains: Lactiplantibacillus plantarum TN10, Lactiplantibacillus plantarum TF2, Pediococcus pentosaceus TF8, Pediococcus acidilactici TE4, and Pediococcus pentosaceus TI6.

**Table 4:** The difference in pH between the sourdoughs fermented with lactic acid bacteria at t_0_ (before sourdough fermentation) and after seven days of fermentation.

Samples	pH	
	Before Sourdough Fermentation	After Sourdough Fermentation
Control	6.320 ± 0.021 ^aA^	6.250 ± 0.035 ^aA^
*Lactiplantibacillus plantarum* TN10 *	5.520 ± 0.503 ^dA^	3.450 ± 0.021 ^dB^
*Lactiplantibacillus plantarum* TF2 *	4.660 ± 0.477 ^cA^	3.470 ± 0.012 ^dB^
*Pediococcus pentosaceus* TF8 *	5.480 ± 0.076 ^cA^	3.530 ± 0.061 ^dcB^
*Pediococcus acidilactici* TE4 *	5.380 ± 0.511 ^cA^	3.620 ± 0.017 ^cB^
*Pediococcus pentosaceus* TI6 *	5.900 ± 0.528 ^dA^	3.830 ± 0.012 ^dB^

* Strains: *Lactiplantibacillus plantarum* TN10, *Lactiplantibacillus plantarum* TF2, *Pediococcus pentosaceus* TF8, *Pediococcus acidilactici* TE4, and *Pediococcus pentosaceus* TI6. A different capital letter means a significant difference between the fermentation times of the same sample. Different lowercase means the significant difference between samples in each fermentation (*p* ≤ 0.05).

**Table 5:** Viability of microorganisms in the sourdoughs fermented with * *Lactiplantibacillus plantarum* TN10, *Lactiplantibacillus plantarum* TF2, *Pediococcus pentosaceus* TF8, *Pediococcus acidilactici* TE4, and *Pediococcus pentosaceus* TI6, and after sourdough drying. Results are expressed as log CFU/g.

Samples	Log (CFU/g)					
	Before Fermentation	After Fermentation	Spray-Dry	Freeze-Dry	Dry at 40 °C	Dry at Low Humidity
Control	ND	ND	ND	ND	ND	ND
*Lactiplantibacillus plantarum* TN10 *	8.85 ± 0.08 ^bB^	8.78 ± 0.04 ^cB^	11.11 ± 0.20 ^aA^	8.85 ± 0.08 ^bcB^	8.27 ± 0.13 ^cC^	8.86 ± 0.09 ^bB^
*Lactiplantibacillus plantarum* TF2 *	8.70 ± 0.13 ^cB^	9.14 ± 0.07 ^bAB^	9.44 ± 0.23 ^dA^	8.70 ± 0.13 ^cB^	6.74 ± 0.09 ^bD^	7.27 ± 0.07 ^cC^
*Pediococcus pentosaceus* TF8 *	8.75 ± 0.04 ^cB^	8.87 ± 0.07 ^cB^	9.49 ± 0.31 ^dA^	8.75 ± 0.04 ^cB^	7.46 ± 0.11 ^cC^	8.92 ± 0.03 ^bB^
*Pediococcus acidilactici* TE4 *	9.19 ± 0.00 ^aC^	10.62 ± 0.08 ^aA^	9.91 ± 0.10 ^cB^	9.19 ± 0.04 ^aC^	7.99 ± 0.07 ^cD^	9.32 ± 0.01 ^aC^
*Pediococcus pentosaceus* TI6 *	9.18 ± 0.063 ^aB^	9.23 ± 0.03 ^bA^	10.33 ± 0.10 ^bB^	9.18 ± 0.06 ^aB^	8.49 ± 0.16 ^cD^	9.50 ± 0.01 ^aB^

ND means non-detectable values. Different lowercase letters mean a significant difference between values in the same row. Different capital letters mean significant differences between the LAB count at the same sampling point (*p* ≤ 0.05).

**Table 6:** The yield of the weight of the different dry sourdoughs is expressed as a residual percentage of the original weight.

Samples	The Yield of the Weight (%)			
	Spray-Dry	Freeze-Dry	Dry at 40 °C	Dry at Low Humidity
Control	4.0 ± 0.2 ^cD^	42.0 ± 0.3 ^edC^	44.0 ± 0.6 ^bcB^	50.0 ± 0.5 ^aA^
*Lactiplantibacillus plantarum* TN10 *	4.0 ±0.3 ^bcD^	43.0 ± 0.2 ^cB^	38.0 ± 0.2 ^dC^	51.0 ± 0.1 ^aA^
*Lactiplantibacillus plantarum* TF2 *	5.0 ± 0.3 ^bD^	42.0 ± 0.5 ^edB^	39.0 ± 0.6 ^dC^	47.0 ± 0.5 ^cA^
*Pediococcus pentosaceus* TF8 *	6.0 ± 0.1 ^aD^	44.0 ± 0.1 ^bC^	45.0 ± 0.2 ^bB^	48.0 ± 0.6 ^bA^
*Pediococcus acidilactici* TE4 *	4.0 ± 0.3 ^bcD^	40.0 ± 0.5 ^edC^	44.0 ± 0.5 ^cB^	47.0 ± 0.3 ^cA^
*Pediococcus pentosaceus* TI6 *	3.0 ± 0.3 ^dD^	46.0 ± 0.2 ^aB^	68.0 ± 0.2 ^aA^	39.0 ± 0.1 ^dC^

* Strains: *Lactiplantibacillus plantarum* TN10, *Lactiplantibacillus plantarum* TF2, *Pediococcus pentosaceus* TF8, *Pediococcus acidilactici* TE4, and *Pediococcus pentosaceus* TI6. Different lowercase letters mean a significant difference in the yield between samples after the same drying technique. Different capital letters mean significant differences in the yield between the drying techniques for the same sample (*p* ≤ 0.05).

**Table 7:** Determination of the organic acids in the dry sourdoughs fermented with lactic acid bacteria.

Samples	g of Lactic Acid/kg of Product			
	Spray-Dry	Freeze-Dry	Dry at 40 °C	Dry at Low Humidity
Control	1.1 ± 0.0 ^eB^	0.6 ± 0.0 ^dA^	0.7 ± 0.0 ^eB^	0.7 ± 0.0 ^eB^
*Lactiplantibacillus plantarum* TN10 *	26.3 ± 0.2 ^aA^	17.3 ± 0.0 ^aB^	7.6 ± 0.3 ^bC^	13.7 ± 0.2 ^aC^
*Lactiplantibacillus plantarum* TF2 *	19.8 ± 0.0 ^bA^	17.4 ± 0.0 ^aB^	14.3 ± 0.0 ^bC^	13.7 ± 0.5 ^aC^
*Pediococcus pentosaceus* TF8 *	17.7 ± 0.0 ^cA^	16.9 ± 0.1 ^aA^	15.7 ± 0.5 ^aB^	12.7 ± 0.0 ^bC^
*Pediococcus acidilactici* TE4 *	20.4 ± 0.7 ^bA^	13.9 ± 0.1 ^bB^	11.6 ± 0.0 ^cC^	11.6 ± 0.0 ^cC^
*Pediococcus pentosaceus* TI6 *	15.7 ± 0.0 ^dA^	10.5 ± 0.5 ^cB^	8.6 ± 0.1 ^dC^	8.5 ± 0.1 ^dC^

* Strains: *Lactiplantibacillus plantarum* TN10, *Lactiplantibacillus plantarum* TF2, *Pediococcus pentosaceus* TF8, *Pediococcus acidilactici* TE4, and *Pediococcus pentosaceus* TI6. Different lowercase letters mean a significant difference in lactic acid concentration between samples at the same drying technique. Different capital letters mean significant differences in the concentration of lactic acid between drying techniques (*p* ≤ 0.05).

## Data Availability

The data are available from the corresponding author.
